# Personality and Islamic religiosity: Preliminary survey data of Bruneian Malay Muslim university students and their psychological well-being, unethical behavior, and dark triad traits

**DOI:** 10.1016/j.dib.2020.105486

**Published:** 2020-04-07

**Authors:** Nur Amali Aminnuddin

**Affiliations:** Academy of Brunei Studies, Universiti Brunei Darussalam, Brunei

**Keywords:** HEXACO, Personality, Islamic religiosity, Psychological well-being, Unethical behavior, Machiavellianism, Narcissism, Psychopathy

## Abstract

The paper presents data collected using measures of personality, Islamic religiosity, psychological well-being, unethical behavior, and dark triad traits. The sample consists 277 Bruneian Malay Muslim university students. The participants were sampled at a public university in Brunei. Through cooperation with faculty members, a link to the online questionnaire was posted on the learning management system platform. The targeted population was informed they were invited to participate voluntarily, and all information would be confidential. Completed questionnaires were submitted by 171 females and 106 males. The age ranged between 18 and 30 years old (*M* = 19.62, *SD* = 1.65). This data article presented the demographic characteristics of participants, followed by descriptive statistics of the measures concerning primarily personality and Islamic religiosity, followed by brief measures of psychological nature specifically psychological well-being, unethical behavior, and dark triad traits. Reliability and correlation details of measures were also provided. The comprehensive data, as detailed out in this paper, has various potential for further research and analyses by researchers who are interested in the measures and data presented.

**Specifications table**

**Table:** 

Subject	Psychology (General); Social Psychology
Specific subject area	Islam; Psychology of Religion; Psychology of Islam; Personality Psychology; I-O Psychology; Individual Differences
Type of data	Table
How data were acquired	Data was acquired through an online cross-sectional survey. A link to the questionnaire was provided to the targeted population.
Data format	Raw
	Cleaned
	Analyzed
Parameters for data collection	Samples consisted of undergraduate students at a public university in Brunei. They were sampled among those who registered for a compulsory core module at the university.
Description of data collection	Proper approval had been acquired for conducting the survey. With cooperation from faculty members, a link to the questionnaire was attached to a notice on the learning management system platform.
Data source location	Brunei
Data accessibility	Data is included with this article, and on Mendeley Data Repository: http://dx.doi.org/10.17632/6r8y5wc2bx.1

## Value of the Data

• The data provides insights into the intricate relationship between personality and Islamic religiosity.
• More specifically, insights of the complex interplay between personality and religiosity can also provide a valuable opportunity to delve deeper into their impacts toward individuals, such as a person's psychological well-being, unethical behavior, and dark triad traits.
• The data also specifies religiosity specifically of the Islamic context with various dimensions that can be examined individually or concurrently together, or in relation with other measures in the data file.
• Due to the nature of the data and its comprehensiveness, various analyses can be conducted including analysis of variance, factor analysis, and structural equation modeling. Although the data only concerns Brunei Muslim university students, it can be used in parallel with data file that contained information from other groups in the country, which may provide comparative findings, or be used as a basis of replication.
• With interdisciplinary and multidisciplinary approaches gaining momentum in the recent years, these data can be useful for researchers from various disciplines, including but not limited to, social psychology, psychology of religion especially of Islam, Islamic studies, management, and sociology. Following further analyses and findings being interpreted, these also to a certain extent have significances for management in organizations and policymakers.

## 1 Data description

The data includes measures of personality and Islamic religiosity, followed by psychological well-being, unethical behavior, and dark triad traits (see “Data” file under supplementary material) through cross-sectional survey (see “Questionnaire” file under supplementary material). Sampled population consisted of a convenience sample of 277 Bruneian Malay Muslim university students from a public university. No information was missing due to all items being made compulsory to be completed prior to submission. The participants were all students who registered for a compulsory core module for all students at the institution. The participants were made up of 171 females and 106 males. The age ranged between 18 and 30 years old (*M* = 19.62, *SD* = 1.65). All of them were Bruneian Malay Muslims, as well as being undergraduate students. Characteristics of age and faculty of students are presented (see Table 1). The data file also contained details on the major taken by students. However, due to the large number of majors, it is not presented here. Overall, due to no missing data, it is easy to replicate the computation as stated earlier. Hence, no syntax is provided in this data paper. Some variables were computed to yield its sum, while others were computed to get its mean value. These were done according to how the measures were employed prior to this. All reverse coded items in the data file had already been coded back to be aligned with other items within the same measure. Hence, no further reverse coding is necessary. Descriptive statistics concerning measures and their reliability are presented (see Table 2). For IBS, reliability analysis was not possible due to all items having no variances except for IBS5, which only two participants responded differently than the others. For correlation details, three tables were presented: personality (see Table 3), Islamic religiosity (see Table 4), and other psychological measures (Table 5).

**Table 1:** Age and Faculty.

Variables	Category	*N*	%
Age	18	50	18.1%
	19	122	44%
	20	59	21.3%
	21	16	5.8%
	22	15	5.4%
	23	4	1.4%
	24	7	2.5%
	25	2	0.7%
	30	2	0.7%
	Total	277	
Faculty	Academy of Brunei Studies	13	4.7%
	Faculty of Arts and Social Sciences	83	30%
	Faculty of Integrated Technologies	5	1.8%
	Faculty of Science	123	44.4%
	Institute of Health Sciences	16	5.8%
	School of Business and Economics	37	13.4%
	Total	277

**Table 2:** Descriptive statistics of measures and reliability.

Measures	Items	Rating	*M*	Reliability
Honesty-Humility	HPIR6, HPIR12R, HPIR18, HPIR24R, HPIR30R, HPIR36, HPIR42R, HPIR48R, HPIR54, HPIR60R	1 to 5	3.52	.62
Emotionality	HPIR5, HPIR11, HPIR17, HPIR23, HPIR29, HPIR35R, HPIR41R, HPIR47, HPIR53R, HPIR59R	1 to 5	3.5	.74
Extraversion	HPIR4, HPIR10R, HPIR16, HPIR22, HPIR28R, HPIR34, HPIR40, HPIR46R, HPIR52R, HPIR58	1 to 5	2.97	.74
Agreeableness	HPIR3, HPIR9R, HPIR15R, HPIR21R, HPIR27, HPIR33, HPIR39, HPIR45, HPIR51, HPIR57R	1 to 5	3.21	.67
Conscientiousness	HPIR2, HPIR8, HPIR14R, HPIR20R, HPIR26R, HPIR32R, HPIR38, HPIR44R, HPIR50, HPIR56R	1 to 5	3.24	.68
Openness to Experiences	HPIR1R, HPIR7, HPIR13, HPIR19R, HPIR25, HPIR31R, HPIR37, HPIR43, HPIR49R, HPIR55R	1 to 5	3.42	.58
IBS	IBS1 to IBS5	0 to 2	–	–
IEPS	IEPS1 to IEPS10	1 to 5	4.37	.89
IUS	IUS1 to IUS4	1 to 5	4.34	.83
IRCS	IRCS1 to IRCS6	1 to 5	4.56	.91
IIS	IIS1 to IIS5	1 to 4	3.37	.88
IPRCS	IPRCS1 to IPRCS7	1 to 4	3.33	.90
PARS	PARS1 to PARS3	1 to 4	2.84	.85
IRSS	IRSS1 to IRSS6	0 to 4	.26	.86
IDS	IDS1 to IDS5	0 to 5	3.59	.75
IOS	IOS1 to IOS5	1 to 4	2.62	.79
IES	IES1, IES2, IES3R, IES4	−4 to 4	2.46	.73
GRS	GRS1, GRS2	1 to 5	3.07	.82
SWLS	SWLS1 to SWLS5	1 to 7	4.05	.80
SPANE (Positive)	SPANE1, SPANE3, SPANE5, SPANE7, SPANE10, SPANE12	1 to 5	3.59	.81
SPANE (Negative)	SPANE2, SPANE4, SPANE6, SPANE8, SPANE9, SPANE11	1 to 5	3.01	.78
FS	FS1 to FS8	1 to 7	4.87	.88
Unethical Behavior	UB1 to UB32	1 to 5	1.65	.96
Machiavellianism	SD3M1 to SD3M9	1 to 5	3.35	.79
Narcissism	SD3N1, SD3N2R, SD3N3 to SD3N5, SD3N6R, SD3N7, SD3N8R, SD3N9	1 to 5	2.52	.72
Psychopathy	SD3P1, SD3P2R, SD3P3 to SD3P6, SD3P7R, SD3P8, SD3P9	1 to 5	2.44	.61

*Note.* Please see Section 2.2.2, Section 2.2.3, Section 2.2.4, and Section 2.2.5 for variable abbreviations.

**Table 3:** Correlation of HEXACO Facets.

	Honesty-Humility	Emotionality	Extraversion	Agreeableness	Conscientiousness	Openness to Experience
Honesty-Humility	1	.029	−0.14*	.33***	.20***	.04
Emotionality		1	−0.12*	−0.31***	−0.05	−0.13*
Extraversion			1	.06	.32***	.03
Agreeableness				1	.25***	.06
Conscientiousness					1	.15*
Openness to Experiences						1

**Table 4:** Correlation of Islamic religiosity.

	IBS	IEPS	IUS	IRCS	IIS	IPRCS	PARS	IRSS	IDS	IOS	IES	GRS
IBS	1	.06	.13*	.04	.06	.07	.05	−0.09	.07	.08	.13*	.06
IEPS		1	.62***	.48***	.48***	.45***	.08	−0.28***	.24***	.19**	.24***	.24***
IUS			1	.62***	.51***	.46***	.14*	−0.31***	.29***	.15*	.28***	.23***
IRCS				1	.51***	.56***	.14	−0.12	.38***	.18*	.07	.21*
IIS					1	.61***	.09	−0.33***	.42***	.20**	.22***	.38***
IPRCS						1	.07	−0.30***	.49***	.16**	.17**	.36***
PARS							1	.12*	−0.01	.28***	.08	.04
IRSS								1	−0.16**	−0.01	−0.32***	−0.12
IDS									1	.17***	.14*	.35***
IOS										1	.09	.21***
IES											1	.07
GRS												1

*Note.* Please see Section 2.2.2 for variable abbreviations.

**Table 5:** Correlation of psychological measures.

	SWLS	SPANE (Positive)	SPANE (Negative)	SPANE (Balance)	FS	Unethical Behavior	Machiavellianism	Narcissism	Psychopathy
SWLS	1	.54***	−0.38***	.55***	.59***	.05	−0.09	.21***	−0.14*
SPANE (Positive)		1	−0.36***	.79***	.57***	−0.02	−0.07	.14*	−0.09
SPANE (Negative)			1	−0.86***	−0.34***	−0.02	.07	−0.07	.15*
SPANE (Balance)				1	.54***	.002	−0.09	.13*	−0.15*
FS					1	.01	−0.07	.31***	−0.17**
Unethical Behavior						1	.16**	.13*	.24***
Machiavellianism							1	.24***	.47***
Narcissism								1	.24***
Psychopathy									1

*Note.* Please see Section 2.2.3, Section 2.2.4, and Section 2.2.5 for variable abbreviations.

## 2 Experimental design, materials, and methods

It is only in recent years that the study of religiosity, specifically Islam, through psychological approach gains momentum in Brunei. Among them, a number had been done in the context of the workplace and Islamic work ethic [1], [2], [3]. However, with the limited number of literature, this is puzzling, considering that Islam has always been considered as the most dominant cultural factor influencing the society in Brunei at all levels, i.e., individuals, society, and the government [4]. Due to this, data was collected to facilitate the effort in addressing this highly potential research area and the current void in the context of Islamic religiosity and Brunei. Hence, this paper contributes for the first time preliminary data on personality and Islamic religiosity of individuals in Brunei, together with other relevant variables of psychological nature. The data file is attached together with the questionnaire used for data collection, in this data article (see “Data” file and “Questionnaire” file under supplementary material). All the items, including labels, values and ratings, are provided and clearly presented in the attached supplementary material.

### 2.1 Data collection

This data article aimed at documenting data on personality and Islamic religiosity that can be examined in tandem with psychological well-being, unethical behavior, and dark triad traits. The data had not been used prior to this paper. Therefore, it has significant value and incentive for researchers to use it in their studies. The parameter of sample population was a Bruneian Malay Muslim university student. The targeted university was Universiti Brunei Darussalam, a public university in the country.

Relevant department at the faculty was contacted to get approval. For convenience, undergraduates who registered for a specific core module that was compulsory for all students were targeted. Following approval, cooperation with faculty members were achieved where they helped to issue a notice on the module's learning management system platform, attaching the link to the online questionnaire. Questionnaires were to be completed anonymously. Participants were also informed that all collected data are confidential, and their participations were anonymously done. In fact, there is no possibility for data to be traced back to a specific individual.

### 2.2 Questionnaire

The questionnaire administered to the sample population is attached here in its original form (see “Questionnaire” file under supplementary material). The questionnaire was created using Google Form. It contained demographic items such as age, gender, religion, ethnicity, university, program, faculty, and major. This ensures that the targeted population remained true to what the parameter of sample population was. After the demographic items, items relevant to measures of personality, Islamic religiosity, psychological well-being, unethical behavior, and dark triad traits were asked.

#### 2.2.1 Personality

To assess personality, the instrument HEXACO-60 was used [5]. With 60 items, it had six facets: honesty-humility, emotionality, extraversion, agreeableness, conscientiousness, and openness to experience. Items in this scale were rated on a 5-point Likert scale (1 = strongly disagree, 2 = disagree, 3 = neutral, 4 = agree, 5 = strongly agree). In the data file, the items were labeled together with running numbers, i.e., HPIR1R to HPIR60R. Those that were reverse coded items had the letter R at the end (for example, HPIR1R). If there was none, it was not a reverse coded item (for example, HPIR2). Items that were reverse coded items were coded back to be aligned with the other items. Hence, no reverse coding is necessary when using this data file. A variable was created representing the mean score for each personality factor in HEXACO, and each was labeled accordingly in the data file: HONESTYHUMILITY, EMOTIONALITY, EXTRAVERSION, AGREEABLENESS, CONSCIENTIOUSNESS, and OPENNESSTOEXPERIENCE.

#### 2.2.2 Islamic religiosity

In order to measure religiosity, the Psychological Measures of Islamic Religiosity was employed [6]. The instrument has several subscales in it. The scales were the 5-item Islamic Belief Subscale (IBS1 to IBS5), the 10-item Islamic Ethical Principles Subscale (IEPS1 to IEPS10), the 4-item Islamic Universality Subscale (IUS1 to IUS4), the 6-item Islamic Religious Conversion Subscale (IRCS1 to IRCS6), the 5-item Islamic Identification Subscale (IIS1 to IIS5), the 7-item Islamic Positive Religious Coping Subscale (IPRCS1 to IPRCS7), the 3-item Punishing Allah Reappraisal Subscale (PARS1 to PARS3), the 6-item Islamic Religious Struggle Subscale (IRSS1 to IRSS6), the 5-item Islamic Duty Subscale (IDS1 to IDS5), the 5-item Islamic Obligation Subscale (IOS1 to IOS5), and the 4-item Islamic Exclusivism Subscale (IES1 to IES4). Furthermore, one scale was attached to this instrument: the Global Religiousness Scale [6]. This was to be used in together with the Psychological Measures of Islamic Religiosity. Following mean computations, these were labeled in the data file respectively as IBS, IEPS, IUS, IRCS, IIS, IPRCS, PARS, IRSS, IDS, IOS, IES, and GRS.

For the 6-item Islamic Religious Conversion Subscale, prior to presenting the items, one statement was presented: “In my life, I have changed from a non-religious person to a religious person.” If a participant selected “No,” then the individual was not asked to complete the 6-item Islamic Religious Conversion Subscale. If a participant selected “Yes,” then the individual will be presented with the subscale.

Concerning the ratings, due to the large number of items with varying ratings attached to them, they will not be presented here. However, the items and their ratings, including for other measures, were provided in the supplementary material, as well as already labeled and assigned properly in the data file (see “Data” file and “Questionnaire” file under supplementary material). The information presented are sufficient for replication purpose.

#### 2.2.3 Psychological well-being

Three measures were used to assess psychological well-being: the 5-item Satisfaction with Life Scale [7], the 12-item Scale of Positive and Negative Experience [8], and the 8-item Flourishing Scale [8]. For the Satisfaction with Life Scale, items were rated on a 7-point Likert scale (1 = strongly disagree, 2 = disagree, 3 = slightly disagree, 4 = neither agree nor disagree, 5 = slightly agree, 6 = agree, 7 = strongly agree). The items were labeled similarly with other instruments, i.e., SWLS1 to SWLS2. The scoring was designed to be the sum of all scores. In the data file, this was labeled as SWLS. The higher it is the more satisfied the person is in life.

For the Scale of Positive and Negative Experience, items were rated on a 5-point Likert scale (1 = very rarely or never, 2 = rarely, 3 = sometimes, 4 = often, 5 = very often or always). The items in the data file were SPANE1 to SPANE12. For positive experience, scores from six items were added up together to yield a variable indicating positive feelings and labeled as SPANEPOSITIVE in the data file. Similarly, with the other six items, it yielded a variable indicating negative feelings, and labeled as SPANENEGATIVE. Another item in the data file was created where SPANEPOSITIVE minus SPANENEGATIVE, resulting to affect balance, which was labeled as SPANEBALANCE.

The same rating for the Satisfaction with Life Scale applied to the Flourishing Scale. The items were labeled as FS1 to FS8. The scoring was the sum of all items, and in the data file this was labeled as FS. The higher the score is, the more the person viewed having psychological resources and strengths.

#### 2.2.4 Unethical behavior

Unethical behavior was assessed using the 32-item Measure of Unethical Behaviors [9]. Items in this instrument were rated on a 5-point Likert scale (1 = very low probability, 2 = low, 3 = average, 4 = high, 5 = very high probability). The items were labeled in the data file as UB1 to UB32. The higher the scoring, the higher the probability of the person engaging in unethical behavior. A variable was created representing the mean score, and this was labeled as UNETHICALBEHAVIOR.

#### 2.2.5 Dark triad traits

To assess the dark triad traits of Machiavellianism, narcissism, and psychopathy, a brief instrument measuring all three was used. The instrument was the 27-item Short Dark Triad [10]. Each trait had 9 items. For example, in the data file, Machiavellianism was labeled as SD3M1 to SD3M9. For narcissism, it was SD3N1 to SD3N9; while it was SD3P1 to SD3P9 for psychopathy. Items in this instrument were rated on a 5-point Likert scale (1 = disagree strongly, 2 = disagree, 3 = neither agree nor disagree, 4 = agree, 5 = agree strongly). There were five items that were reverse coded items. They were already coded back to be aligned with the other items. Hence, no reverse coding is necessary when using this data file. Higher score represents having higher tendency of the dark triad trait. Similar to unethical behavior, a variable was created in the data file representing the mean score for each dark trait. This resulted to the variable MACHIAVELLIANISM, NARCISSISM, and PSYCHOPATHY in the data file.

## Appendix Supplementary materials

Image, application 1

Image, application 2
