# Supplementary material for: Personality and Islamic religiosity: Preliminary survey data of Bruneian Malay Muslim university students and their psychological well-being, unethical behavior, and dark triad traits
Source: Data Brief. 2020 Apr 7;30:105486. doi: 10.1016/j.dib.2020.105486 (PMC7160428; doi:10.1016/j.dib.2020.105486)
Supplement: Supplementary file 1 [file mmc1.pdf]

Questionnaire

* Required

1. Age *

2. Gender *

   Mark only one oval.

   - Male
   - Female

3. Religion. *

   Mark only one oval.

   - Islam
   - Other:

4. Ethnicity. *

   Mark only one oval.

   - Malay
   - Other:

5. University *

   Mark only one oval.

   - Universiti Brunei Darussalam
   - Universiti Teknologi Brunei
   - Universiti Islam Sultan Sharif Ali
   - Other:

6. Current level of study *

   Mark only one oval.

   - Bachelor's degree
   - Master's degree
   - PhD
   - Other:

7. Faculty *

8. What do you major in? If do not have a major yet, do specify what you intend to major in. Example: Degree in accounting. *

HEXACO Personality Inventory

9. Please read each statement and decide how much you agree or disagree with that statement. *

Mark only one oval per row.

	Strongly disagree	Disagree	Neutral	Agree	Strongly agree
I would be quite bored by a visit to an art gallery.	◯	◯	◯	◯	◯
I plan ahead and organize things, to avoid scrambling at the last minute.	◯	◯	◯	◯	◯
I rarely hold a grudge, even against people who have badly wronged me.	◯	◯	◯	◯	◯
I feel reasonably satisfied with myself overall.	◯	◯	◯	◯	◯
I would feel afraid if I had to travel in bad weather conditions.	◯	◯	◯	◯	◯
I wouldn't use flattery to get a raise or promotion at work, even if I thought it would succeed.	◯	◯	◯	◯	◯
I'm interested in learning about the history and politics of other countries.	◯	◯	◯	◯	◯
I often push myself very hard when trying to achieve a goal.	◯	◯	◯	◯	◯
People sometimes tell me that I am too critical of others.	◯	◯	◯	◯	◯
I rarely express my opinions in group meetings.	◯	◯	◯	◯	◯

10. Please read each statement and decide how much you agree or disagree with that statement. *

Mark only one oval per row.

	Strongly disagree	Disagree	Neutral	Agree	Strongly agree
I sometimes can't help worrying about little things.	◯	◯	◯	◯	◯
If I knew that I could never get caught, I would be willing to steal a million dollars.	◯	◯	◯	◯	◯
I would enjoy creating a work of art, such as a novel, a song, or a painting.	◯	◯	◯	◯	◯
When working on something, I don't pay much attention to small details.	◯	◯	◯	◯	◯
People sometimes tell me that I'm too stubborn.	◯	◯	◯	◯	◯
I prefer jobs that involve active social interaction to those that involve working alone.	◯	◯	◯	◯	◯
When I suffer from a painful experience, I need someone to make me feel comfortable.	◯	◯	◯	◯	◯
Having a lot of money is not especially important to me.	◯	◯	◯	◯	◯
I think that paying attention to radical ideas is a waste of time.	◯	◯	◯	◯	◯
I make decisions based on the feeling of the moment rather than on careful thought.	◯	◯	◯	◯	◯

11. Please read each statement and decide how much you agree or disagree with that statement. *

Mark only one oval per row.

	Strongly disagree	Disagree	Neutral	Agree	Strongly agree
People think of me as someone who has a quick temper.	◯	◯	◯	◯	◯
On most days, I feel cheerful and optimistic.	◯	◯	◯	◯	◯
I feel like crying when I see other people crying.	◯	◯	◯	◯	◯
I think that I am entitled to more respect than the average person is.	◯	◯	◯	◯	◯
If I had the opportunity, I would like to attend a classical music concert.	◯	◯	◯	◯	◯
When working, I sometimes have difficulties due to being disorganized.	◯	◯	◯	◯	◯
My attitude toward people who have treated me badly is “forgive and forget”.	◯	◯	◯	◯	◯
I feel that I am an unpopular person.	◯	◯	◯	◯	◯
When it comes to physical danger, I am very fearful.	◯	◯	◯	◯	◯
If I want something from someone, I will laugh at that person's worst jokes.	◯	◯	◯	◯	◯

12. Please read each statement and decide how much you agree or disagree with that statement. *

Mark only one oval per row.

	Strongly disagree	Disagree	Neutral	Agree	Strongly agree
I’ve never really enjoyed looking through an encyclopedia.	◯	◯	◯	◯	◯
I do only the minimum amount of work needed to get by.	◯	◯	◯	◯	◯
I tend to be lenient in judging other people.	◯	◯	◯	◯	◯
In social situations, I’m usually the one who makes the first move.	◯	◯	◯	◯	◯
I worry a lot less than most people do.	◯	◯	◯	◯	◯
I would never accept a bribe, even if it were very large.	◯	◯	◯	◯	◯
People have often told me that I have a good imagination.	◯	◯	◯	◯	◯
I always try to be accurate in my work, even at the expense of time.	◯	◯	◯	◯	◯
I am usually quite flexible in my opinions when people disagree with me.	◯	◯	◯	◯	◯
The first thing that I always do in a new place is to make friends.	◯	◯	◯	◯	◯

13. Please read each statement and decide how much you agree or disagree with that statement. *

Mark only one oval per row.

	Strongly disagree	Disagree	Neutral	Agree	Strongly agree
I can handle difficult situations without needing emotional support from anyone else.	⬭	⬭	⬭	⬭	⬭
I would get a lot of pleasure from owning expensive luxury goods.	⬭	⬭	⬭	⬭	⬭
I like people who have unconventional views.	⬭	⬭	⬭	⬭	⬭
I make a lot of mistakes because I don't think before I act.	⬭	⬭	⬭	⬭	⬭
Most people tend to get angry more quickly than I do.	⬭	⬭	⬭	⬭	⬭
Most people are more upbeat and dynamic than I generally am.	⬭	⬭	⬭	⬭	⬭
I feel strong emotions when someone close to me is going away for a long time.	⬭	⬭	⬭	⬭	⬭
I want people to know that I am an important person of high status.	⬭	⬭	⬭	⬭	⬭
I don't think of myself as the artistic or creative type.	⬭	⬭	⬭	⬭	⬭
People often call me a perfectionist.	⬭	⬭	⬭	⬭	⬭

14. Please read each statement and decide how much you agree or disagree with that statement. *

Mark only one oval per row.

	Strongly disagree	Disagree	Neutral	Agree	Strongly agree
Even when people make a lot of mistakes, I rarely say anything negative.	⬭	⬭	⬭	⬭	⬭
I sometimes feel that I am a worthless person.	⬭	⬭	⬭	⬭	⬭
Even in an emergency I wouldn't feel like panicking.	⬭	⬭	⬭	⬭	⬭
I wouldn't pretend to like someone just to get that person to do favors for me.	⬭	⬭	⬭	⬭	⬭
I find it boring to discuss philosophy.	⬭	⬭	⬭	⬭	⬭
I prefer to do whatever comes to mind, rather than stick to a plan.	⬭	⬭	⬭	⬭	⬭
When people tell me that I'm wrong, my first reaction is to argue with them.	⬭	⬭	⬭	⬭	⬭
When I'm in a group of people, I'm often the one who speaks on behalf of the group.	⬭	⬭	⬭	⬭	⬭
I remain unemotional even in situations where most people get very sentimental.	⬭	⬭	⬭	⬭	⬭
I'd be tempted to use counterfeit money, if I were sure I could get away with it.	⬭	⬭	⬭	⬭	⬭

YOU COMPLETED ALMOST HALF OF THE SURVEY! KEEP IT UP!

Psychological Well-Being

Satisfaction With Life Scale (SWLS)

15. Please read each statement and decide how much you agree or disagree with that statement. *

Mark only one oval per row.

	Strongly disagree	Disagree	Slightly disagree	Neither agree nor disagree	Slightly agree	Agree	Strongly agree
In most ways my life is close to my ideal.	⬭	⬭	⬭	⬭	⬭	⬭	⬭
The conditions of my life are excellent.	⬭	⬭	⬭	⬭	⬭	⬭	⬭
I am satisfied with my life.	⬭	⬭	⬭	⬭	⬭	⬭	⬭
So far I have gotten the important things I want in life.	⬭	⬭	⬭	⬭	⬭	⬭	⬭
If I could live my life over, I would change almost nothing.	⬭	⬭	⬭	⬭	⬭	⬭	⬭

Scale of Positive and Negative Experience(SPANE)

16. Please think about what you have been doing and experiencing during the past four weeks. Then report how much you experienced each of the following feelings, using the scale below. *

Mark only one oval per row.

	Very Rarely or Never	Rarely	Sometimes	Often	Very Often or Always
Positive	⬭	⬭	⬭	⬭	⬭
Negative	⬭	⬭	⬭	⬭	⬭
Good	⬭	⬭	⬭	⬭	⬭
Bad	⬭	⬭	⬭	⬭	⬭
Pleasant	⬭	⬭	⬭	⬭	⬭
Unpleasant	⬭	⬭	⬭	⬭	⬭
Happy	⬭	⬭	⬭	⬭	⬭
Sad	⬭	⬭	⬭	⬭	⬭
Afraid	⬭	⬭	⬭	⬭	⬭
Joyful	⬭	⬭	⬭	⬭	⬭
Angry	⬭	⬭	⬭	⬭	⬭
Contented	⬭	⬭	⬭	⬭	⬭

Flourishing Scale (FS)

17. Please read each statement and decide how much you agree or disagree with that statement. *

Mark only one oval per row.

	Strongly disagree	Disagree	Slightly disagree	Neither agree nor disagree	Slightly agree	Agree	Strongly agree
I lead a purposeful and meaningful life.	⬭	⬭	⬭	⬭	⬭	⬭	⬭
My social relationships are supportive and rewarding.	⬭	⬭	⬭	⬭	⬭	⬭	⬭
I am engaged and interested in my daily activities.	⬭	⬭	⬭	⬭	⬭	⬭	⬭
I actively contribute to the happiness and well-being of others.	⬭	⬭	⬭	⬭	⬭	⬭	⬭
I am competent and capable in the activities that are important to me.	⬭	⬭	⬭	⬭	⬭	⬭	⬭
I am a good person and live a good life.	⬭	⬭	⬭	⬭	⬭	⬭	⬭
I am optimistic about my future.	⬭	⬭	⬭	⬭	⬭	⬭	⬭
People respect me.	⬭	⬭	⬭	⬭	⬭	⬭	⬭

Unethical Behavior

18. There are many hypothetical vignettes (scenarios) for activities at work. Some vignettes may not be applicable to your situation. If you were in that situation, what is the probability that you will take action as suggested in the vignette? *

Mark only one oval per row.

	Very Low Probability	Low	Average	High	Very high probability
Use office supplies (paper, pen), Xerox machine, and stamps for personal purposes.	⬭	⬭	⬭	⬭	⬭
Make personal long-distance (mobile phone) calls at work.	⬭	⬭	⬭	⬭	⬭
Waste company time surfing on the Internet, playing computer games, and socializing.	⬭	⬭	⬭	⬭	⬭
Abuse the company expense accounts and falsify accounting records.	⬭	⬭	⬭	⬭	⬭
Fly first class and spend a lot of company money on a business trip.	⬭	⬭	⬭	⬭	⬭
Borrow $20 from a cash register overnight without asking.	⬭	⬭	⬭	⬭	⬭
Take merchandise and/or cash home.	⬭	⬭	⬭	⬭	⬭
Give merchandise away to personal friends (no charge to the customers).	⬭	⬭	⬭	⬭	⬭
Overcharge customers to increase sales and to earn higher bonus.	⬭	⬭	⬭	⬭	⬭
Trick people to buy new or additional services.	⬭	⬭	⬭	⬭	⬭

19. *

Mark only one oval per row.

	Very low probability	Low	Average	High	Very high probability
Advise customers for unnecessary repairs and services.	⬭	⬭	⬭	⬭	⬭
Give customers “discounts” first and then charge them more money later (bait & switch).	⬭	⬭	⬭	⬭	⬭
Make more money by deliberately not letting clients know about their benefits.	⬭	⬭	⬭	⬭	⬭
Use low quality inputs in producing products and services and keep profits high.	⬭	⬭	⬭	⬭	⬭
Reduce quantity of materials in selling the same brand name product (14 oz. in a 16 oz. can).	⬭	⬭	⬭	⬭	⬭
Use creative accounting to hide the company’s true financial status and increase personal gain.	⬭	⬭	⬭	⬭	⬭
Manipulate stock prices to earn big personal bonus.	⬭	⬭	⬭	⬭	⬭
Give misleading information to investors for short-term personal profits.	⬭	⬭	⬭	⬭	⬭
Give large contracts/projects to personal friends and relatives.	⬭	⬭	⬭	⬭	⬭
Bribe government officials in a foreign country to win big contracts and personal bonus.	⬭	⬭	⬭	⬭	⬭

20. *

Mark only one oval per row.

	Very low probability	Low	Average	High	Very high probability
Receive gifts, money, and loans (bribery) from others due to one’s position and power.	⬭	⬭	⬭	⬭	⬭
Lay off 500 employees to save the company money and increase one’s personal bonus.	⬭	⬭	⬭	⬭	⬭
Use employees’ pension for personal (self-interest) purposes.	⬭	⬭	⬭	⬭	⬭
Decline reasonable claims by others to avoid company’s expenses.	⬭	⬭	⬭	⬭	⬭
Delay payment to make the most use of the organization’s money.	⬭	⬭	⬭	⬭	⬭
Pressure suppliers to reduce prices.	⬭	⬭	⬭	⬭	⬭
Reveal company secrets (proprietary information) for several million dollars.	⬭	⬭	⬭	⬭	⬭
Offer low prices to customers and force small businesses in the downtown area to close.	⬭	⬭	⬭	⬭	⬭
Offer “no” severance payments to laid off employees (not required by law in the U.S.).	⬭	⬭	⬭	⬭	⬭
Take no action for shoplifting by customers.	⬭	⬭	⬭	⬭	⬭
Take no action for employees who steal cash/merchandise.	⬭	⬭	⬭	⬭	⬭
Take no action for the fraudulent charges by one’s company.	⬭	⬭	⬭	⬭	⬭

Are you a Muslim?

21. Are you a Muslim? *

Mark only one oval.

- ⬭ Yes *Skip to question 22*
- ⬭ No *Skip to question 42*

Psychological Measure of Islamic Religiousness (PMIR)

Islamic Beliefs Subscale

22. Please select the answer that best indicates your reaction to each of the following statements. *

Mark only one oval per row.

	No	Uncertain	Yes
I believe in the existence of Allah	⬭	⬭	⬭
I believe in the Day of Judgment	⬭	⬭	⬭
I believe in the existence of paradise and hell	⬭	⬭	⬭
I believe in the existence of the angels, the Jinn, and Satan	⬭	⬭	⬭
I believe in all the prophets that Allah sent and in the sacred texts that were revealed to them	⬭	⬭	⬭

Islamic Ethical Principles Subscale

23. Please select the answer that best indicates your reaction to each of the following statements. *

Mark only one oval per row.

	Strongly disagree	Disagree	Neutral	Agree	Strongly agree
Because of Islam, I strive to be a humble person	⬭	⬭	⬭	⬭	⬭
Because of Islam, I do my best to honor my parents	⬭	⬭	⬭	⬭	⬭
Because of Islam, I try to help my relatives and neighbors	⬭	⬭	⬭	⬭	⬭
Because of Islam, I try to help the needy and the orphans	⬭	⬭	⬭	⬭	⬭
Because of Islam, I strive to be a tolerant person	⬭	⬭	⬭	⬭	⬭
Because of Islam, I refrain from eating pork	⬭	⬭	⬭	⬭	⬭
Because of Islam, I refrain from drinking alcohol	⬭	⬭	⬭	⬭	⬭
Because of Islam, I refrain from having sex before marriage or outside marriage	⬭	⬭	⬭	⬭	⬭
Because of Islam, I do not consider committing suicide	⬭	⬭	⬭	⬭	⬭
Because of Islam, I refrain from gossip	⬭	⬭	⬭	⬭	⬭

Islamic Universality Subscale

24. Please select the answer that best indicates your reaction to each of the following statements. *

Mark only one oval per row.

	Strongly disagree	Disagree	Neutral	Agree	Strongly agree
I consider every Muslim in the world as my brother or sister	⬭	⬭	⬭	⬭	⬭
I empathise with the suffering of every Muslim in the world	⬭	⬭	⬭	⬭	⬭
One of my major sources of pride is being a Muslim	⬭	⬭	⬭	⬭	⬭
I believe that brotherhood and sisterhood is one the basic tenets of Islam	⬭	⬭	⬭	⬭	⬭

Islamic Religious Conversion Subscale

25. Please answer yes or no to the following statement. *

Mark only one oval per row.

	No	Yes
In my life, I have changed from a non-religious person to a religious person	⬭	⬭

26. If your answer to the above statement is yes, please select the answer that best indicates your reaction to each of the following statements. If you selected no, continue to Islamic Identification Subscale.

Mark only one oval per row.

	Strongly disagree	Disagree	Neutral	Agree	Strongly agree
Becoming more involved in Islam was a turning point in my life	⬭	⬭	⬭	⬭	⬭
Islam has moved from the outside to the very center of my life	⬭	⬭	⬭	⬭	⬭
At one point in my life, I realized that Islam is the solution to all of my problems	⬭	⬭	⬭	⬭	⬭
All at once, I felt that my life has no meaning without Islam	⬭	⬭	⬭	⬭	⬭
All at once, I felt that I am on the wrong path and that I should follow the path of Allah	⬭	⬭	⬭	⬭	⬭
In comparison to the way I used to be, Islam touches every aspect of my life	⬭	⬭	⬭	⬭	⬭

Islamic Identification Subscale

27. Please select the answer that best indicates your reaction to each of the following statements. *

Mark only one oval per row.

	Not at all true	Usually not true	Usually true	Very true	Not Applicable
I pray because I enjoy it	◯	◯	◯	◯	◯
I pray because I find it satisfying	◯	◯	◯	◯	◯
I read the Holy Quran because I feel that Allah is talking to me when I do that	◯	◯	◯	◯	◯
I read the Holy Quran because I find it satisfying	◯	◯	◯	◯	◯
I fast in Ramadan because when I fast I feel close to Allah	◯	◯	◯	◯	◯

Islamic Positive Religious Coping Subscale

28. Please select the answer that best indicates your reaction to each of the following statements. *

Mark only one oval per row.

	I do not do this at all	I do this a little	I do this a medium amount	I do this a lot
When I face a problem in life, I look for a stronger connection with Allah	◯	◯	◯	◯
When I face a problem in life, I consider that a test from Allah to deepen my belief	◯	◯	◯	◯
When I face a problem in life, I seek Allah's love and care	◯	◯	◯	◯
When I face a problem in life, I read the Holy Qura'n to find consolation	◯	◯	◯	◯
When I face a problem in life, I ask for Allah's forgiveness	◯	◯	◯	◯
When I face a problem in life, I remind myself that Allah commanded me to be patient	◯	◯	◯	◯
When I face a problem in life, I do what I can and put the rest in Allah's hands	◯	◯	◯	◯

Punishing Allah Reappraisal Subscale

29. Please select the answer that best indicates your reaction to each of the following statements. *

Mark only one oval per row.

	I do not do this at all	I do this a little	I do this a medium amount	I do this a lot
When I face a problem in life, I believe that I am being punished by Allah for bad actions i did.	◯	◯	◯	◯
When I face a problem in life, I wonder what I did for Allah to punish me	◯	◯	◯	◯
When I face a problem in life, I feel punished by Allah for my lack of devotion	◯	◯	◯	◯

Islamic Religious Struggle Subscale

30. Please select the answer that best indicates your reaction to each of the following statements. *

Mark only one oval per row.

	Never	Rarely	Sometimes	Often	Very often
I find myself doubting the existence of Allah	◯	◯	◯	◯	◯
I find some aspects of Islam to be unfair	◯	◯	◯	◯	◯
I find myself doubting the existence of afterlife	◯	◯	◯	◯	◯
I think that Islam does not fit the modern time	◯	◯	◯	◯	◯
I doubt that the Holy Qur'an is the exact words of Allah	◯	◯	◯	◯	◯
I feel that Islam makes people intolerant	◯	◯	◯	◯	◯

Islamic Duty Subscale

Please select the answer that best indicates your reaction to each of the following statements.

31. How often do you pray? *

Mark only one oval.

- ◯ Never
- ◯ A few times a year
- ◯ Several times a month
- ◯ Several times a week
- ◯ Most of the times the 5 daily prayers
- ◯ Five times a day (5 daily prayers) or more including the Sunnah Prayer

32. How often do you fast? *

Mark only one oval.

- ◯ Never
- ◯ Few times in life
- ◯ Few days of the month of Ramadan each year
- ◯ Half of the month of Ramadan each year
- ◯ The whole month of Ramadan each year
- ◯ Other religious days or sunnah fasts in addition to Ramadan

33. How often do you go to the masjid? *

Mark only one oval.

- ◯ Never
- ◯ A few times in my life
- ◯ A few times a year
- ◯ A few times a month
- ◯ About once or twice a week
- ◯ Once a day or more

34. Except in prayers, how often do you read or listen to the Holy Quran? *

Mark only one oval.

- Never
- A few times in my life
- A few times a year
- Few times a month
- About once or twice a week
- Once a day or more

35. Except in prayers, how often do you engage in zikir or tasbih? *

Mark only one oval.

- Never
- A few times in my life
- A few times in a year
- A few times a month
- About once or twice a week
- Once a day or more

Islamic Obligation Subscale

Please select the answer that best indicates your reaction to each of the following statements.

36. Please select the answer that best indicates your reaction to each of the following statements. *

Mark only one oval per row.

	Not all true	Usually not true	usually true	Very true	Not applicable
I fast in Ramadan because I would feel bad if I did not					
I pray because if I do not, Allah will disapprove of me					
I read the Holy Quran because I would feel guilty if I did not					
I go to the masjid because one is supposed to go to the masjid					
I go to the masjid because others would disapprove of me if I did not					

Islamic Exclusivism Subscale

Please select the answer that best indicates your reaction to each of the following statements.

37. Islam is Allah's complete, unfailing guide to happiness and salvation, which must be totally followed *

Mark only one oval.

- Very strongly disagree
- Strongly disagree
- Moderately disagree
- Slightly disagree
- Slightly agree
- Moderately agree
- Strongly agree
- Very strongly agree

38. Of all the people on this earth, Muslims have a special relationship with Allah because they believe the most in His revealed truths and try the hardest to follow His laws. *

Mark only one oval.

- Very strongly disagree
- Strongly disagree
- Moderately disagree
- Slightly disagree
- Slightly agree
- Moderately agree
- Strongly agree
- Very strongly agree

39. It is more important to be a good person than to believe in Allah and the right religion *

Mark only one oval.

- Very strongly disagree
- Strongly disagree
- Moderately disagree
- Slightly disagree
- Slightly agree
- Moderately agree
- Strongly agree
- Very strongly agree

40. Islam is the best way to worship Allah, and should never be compromised *

Mark only one oval.

- Very strongly disagree
- Strongly disagree
- Moderately disagree
- Slightly disagree
- Slightly agree
- Moderately agree
- Strongly agree
- Very strongly agree

Global Religiousness

41. Please select the answer that best indicates your reaction to each of the following statements. *

Mark only one oval per row.

	Very low	Low	Average	High	Very high
How do you describe your religiousness?					
How do you describe your spirituality?					

The Short Dark Triad (SD3)

Machiavellianism

42. Please indicate how much you agree with each of the following statements. *

Mark only one oval per row.

	Disagree strongly	Disagree	Neither agree nor disagree	Agree	Agree strongly
It's not wise to tell your secrets.					
I like to use clever manipulation to get my way.					
Whatever it takes, you must get the important people on your side					
Avoid direct conflict with others because they may be useful in the future					
It's wise to keep track of information that you can use against people later					
You should wait for the right time to get back at people.					
There are things you should hide from other people to preserve your reputation					
Make sure your plans benefit yourself, not others.					
Most people can be manipulated.					

Narcissism

43. Please indicate how much you agree with each of the following hypothetical statements. *

Mark only one oval per row.

	Disagree strongly	Disagree	Neither agree nor disagree	Agree	Agree strongly
People see me as a natural leader.					
I hate being the center of attention.					
Many group activities tend to be dull without me.					
I know that I am special because everyone keeps telling me so.					
I like to get acquainted with important people.					
I feel embarrassed if someone compliments me.					
I have been compared to famous people.					
I am an average person.					
I insist on getting the respect I deserve.					

Psychopathy

44. Please indicate how much you agree with each of the following hypothetical statements. *

Mark only one oval per row.

	Disagree strongly	Disagree	Neither agree nor disagree	Agree	Agree strongly
I like to get revenge on authorities.	⬭	⬭	⬭	⬭	⬭
I avoid dangerous situations.	⬭	⬭	⬭	⬭	⬭
Payback needs to be quick and nasty.	⬭	⬭	⬭	⬭	⬭
People often say I'm out of control.	⬭	⬭	⬭	⬭	⬭
It's true that I can be mean to others.	⬭	⬭	⬭	⬭	⬭
People who mess with me always regret it.	⬭	⬭	⬭	⬭	⬭
I have never gotten into trouble with the law.	⬭	⬭	⬭	⬭	⬭
I enjoy having sex with people I hardly know	⬭	⬭	⬭	⬭	⬭
I'll say anything to get what I want.	⬭	⬭	⬭	⬭	⬭

This content is neither created nor endorsed by Google.

Google Forms
